# Autism in Developing Countries: Lessons from Iran

**DOI:** 10.1155/2011/145359

**Published:** 2011-12-25

**Authors:** Sayyed Ali Samadi, Roy McConkey

**Affiliations:** Institute of Nursing Research, University of Ulster, Newtownabbey BT37 0QB, UK

## Abstract

Most research into Autism Spectrum Disorders has been conducted in affluent English-speaking countries which have extensive professional support services. This paper describes a series of investigations that was undertaken in Iran, and these findings, together with reviews of research in other low-income countries, are used to identify key lessons in three areas of service provision of particular relevance to developing countries with scarce professional resources: first, the issues to be considered in establishing the prevalence of the condition nationally; second, identification of parental understanding of ASD and the impact it has on them as carers; third, the education and training that could be provided to families when professional supports are sparse. It is concluded that culturally sensitive, parental support strategies must be central to the planning and development of services. Moreover, future research should further elucidate the needs of families and evaluate the impact of culturally tailored interventions designed to promote the children's development and overall family quality of life.

## 1. Introduction

As with most other intellectual and developmental disabilities, Autism Spectrum Disorders (ASD) occurs in every nation of the world, with families often providing life-long care to their affected relative. Unfortunately, in less affluent countries, there is a dearth of studies to inform the development of support services. Three fundamental questions need to be addressed as suggested by the World Health Organisation [1]. What is the likely prevalence of the condition within the country? What impact does a child with ASD have on the family given that they will be most affected? What training and support is beneficial to families so that they can promote the child's development and well-being?

Past research on children with ASD and the effects on parents of having a child with ASD has been limited largely to families in western countries [2]. Nearly all prevalence studies reviewed by Fombonne [3, 4] and Williams et al. [5] were undertaken in Western countries or Asian affluent countries such as Japan. There is limited information on the identification of children with this condition in non-Western, less affluent countries where services for children with any form of special needs are less developed. This shortage of information has raised the unwarranted assumption made by some writers, that autism is rare in non-Western cultures [6, 7]. 

For those families in low-and middle-income countries who have a child with ASD, access to professional support services will be limited. But even so there is a growing recognition of the need for cultural sensitivity in importing knowledge and practices from one culture—such as European nations—into societies with very different cultural backgrounds [8]. For example, different cultures can have different opinions about appropriate intervention and treatment of children with disabilities [9]. Hence indigenous research is needed to identify the particular needs of families in nonwestern countries and how information and supports can be better tailored to their needs and be respectful of their cultures. Indeed for the foreseeable future, it is likely that families rather than professional workers will provide much of the specialist care which children with ASD require [1]. On a positive note, national organisations for children and families with autism now exist in different countries, suggesting that regardless the level of the development of the countries, at least the diagnostic category has travelled around the world. The formation of the World Autism Organisation is a welcome step towards international capacity building.

The paper describes an approach used in Iran to learn more about ASD and its impact on families. The findings from a series of research studies undertaken by the authors are summarised and comparisons are made with the results reported in similar studies undertaken in other countries. Our aim is to identify important lessons that can guide the development of family support services in low-income countries.

By way of background, we begin by summarising the main features of the country. The Islamic Republic of Iran, formerly known as Persia, is located in the Middle East. The capital city is Tehran. It is a vast country of 1.65 million sq km, extending in the north from the Caspian Sea to the Persian Gulf, Strait of Hormuz, and Oman Sea in the south, and from Afghanistan and Pakistan in the east to Iraq and Turkey in the west. Persians (51% population) are the largest ethnic group in the Republic. The main minorities are Azeri (24%), Gilaki and Mazandarani (8%), and Kurds (7%). People are mainly Muslims (89% Shi'a Muslims and 9% Sunni Muslims). Iran became an Islamic Republic in 1979 and is divided into 31 provinces, each of which is headed by a governor-general appointed by the Minister of the Interior.

Children with ASD will usually be diagnosed by medical doctors either privately or through child and family clinics provided by voluntary organisations. State-funded special schools are provided through the Iranian Special Education Organisation although many parents may opt for private schooling. In addition, parents will arrange private therapy for their children. For children more severely affected or with other conditions such as intellectual disability, day centre placements are available through the Iranian State Welfare Organisation. However, these services are only available in larger cities and probably only for more affluent families. Provision for adult services is mostly through private or voluntary organisations that also rely heavily on parental fees.

The programme of research undertaken in Iran focussed on three key areas. First, identifying children with Autism and establishing its prevalence as a driver to the development of service provision. The second focus was on determining the support needs of families as they are likely to be the main means of promoting the children's development and well-being given the dearth of appropriate professional supports found in most developing countries. Thirdly, we aimed to explore the impact that short education and training opportunities for parents can have on their personal well-being.

## 2. Prevalence of Autism in Developing Countries

Although it may be difficult, obtaining information on the identification of children with ASD in different countries and cultures serves a number of uses. First, it can alert governments to the need to adapt or extend education and other services to meet the particular needs of these children and their families in line with the population requirements. Second international comparisons of prevalence may confirm the extent to which the identification of this condition is affected by cultural influences [4] as well as etiological factors or a combination of both. To date studies on cultural factors and ASD have received little attention [10]. Third suitable procedures for identifying children with this condition in particular cultures can be tested rather than presuming that assessment tools developed in other countries will be adequate [11].

### 2.1. Prevalence in Nonwestern Countries

To date nearly all prevalence studies of ASD included in recent reviews [3, 4, 9] were undertaken in developed countries. There is limited information on the identification of children with this condition in developing countries in which services for children with special needs are less developed. Fombonne [3] reported only one nonwestern study from Indonesia which reported a prevalence of 11.7 per 10,000 within a birth cohort who were born between June 1984 and May 1991 [12].

A subsequent study in China with 7,345 children, aged 2–6 years, also reported a prevalence of 11.0 per 10,000 children [13] whereas a study [14] using government population statistics noted an estimated prevalence of 7.9 per 10,000 for children under five years in Hong Kong in the period 2001–2005. However, in those years, there was an overall prevalence in excess of 25 per 10,000 for all children less than 15 years old.

In Iran, Samadi et al. [15] undertook a study based on the data held by the Special Education Organisation on the national screening of five-year-old children for ASD prior to school entry. Out of 1.32 million, five-year-old Iranian children who went through the screening programme for autism using an Iranian translation of the Social Communication Questionnaire (SCQ) [16, 17] over three academic years, 3181 [24.09 per 10,000] were suspected as having autism. However, the number of the children who subsequently were given a diagnosis of autistic disorder when formally assessed using the Persian translation of the Autism Diagnostic Interview [ADI-R] [18, 19] was around one quarter of this total *N* = 826 [6.26 per 10,000]. In all three years, more boys than girls were suspected as having autism or were diagnosed with it. The proportions of around 4 : 1 are comparable to studies in other countries. A similar disparity is also reflected in the prevalence for boys and girls, with an average for boys of close to 10 per 10,000 but of 2.4 per 10,000 for girls. It was also found that the prevalence of children assessed as having ASD was twice as high in the more developed Provinces of Iran (8.81 per 10,000) than in the less developed Provinces (3.88 per 10,000), a finding also found in other countries [9].

The overall Iranian prevalence of 6.26 per 10,000 for five year olds is similar to that previously reported for certain European countries and for Hong Kong, as noted earlier. Nonetheless, the reported Iranian rates are much lower than those reported for Sweden [20], USA [21], and England [22] with rates of up to 40 per 10,000 for children with autistic disorder.

### 2.2. Reasons for Lower Prevalence

Various factors might explain the lower prevalence of autism in less developed countries such as Iran. In Iranian culture, a diagnosis of disability is likely to be seen as stigmatizing [23]. As parents are keen for their child to attend schools for ordinary developing students rather than being referred to special schools, they may underreport the child's difficulties to assessors even though they are aware of them. The screening tools that are used in Iran (as well as other countries) rely heavily on parental reports with limited time and opportunity for assessors to observe and interact with the child and for them to make consensus decisions [22]. By contrast, in Western countries parents may be eager to obtain a diagnosis for their child's difficulties as this enables them to access additional services, whereas these are not readily available in Iran. On the other hand, Iranian parents will voluntarily seek out a diagnosis when they recognise the severity of their child's problems [24].

A second reason for lower prevalence rates is that children who have associated conditions such as intellectual disabilities and epilepsy may have been diverted from educational services at an earlier age and, therefore, are not included in the screening for pupils enrolling in elementary schools. In addition, childhood mortality for more severely affected children could be greater, especially in poorer areas [25]. All these factors would reduce the prevalence of ASD compared to Western countries with their more developed health and education services.

It is possible that different child-rearing practices, adult tolerance, and expectations around children's behaviours could be other reasons for the difference in the prevalence rates of autism in Iran. Iranian culture and families may be more tolerant of behaviours in children that in western societies would be seen as “abnormal”, although those children presenting for clinical assessments tended to show the same symptoms as documented in other studies [24]. de Giacomo and Fombonne [26] found that the most common parental concerns were for delay in speech and language development, followed by abnormal signs of socioemotional behaviour and medical problems or delay in reaching milestones. In contrast, Daley [2] reported that Indian parents rated social difficulties such as lack of interest in people, poor eye contact, and showing no interest in playing with other children as their primary concern.

Also the culture may not have the same environmental influences that trigger ASD-like behaviours in children. A study compared three groups of children with ASD from South Korean, Korean American, and American families to explore the influences of culture with reference to the behaviours of autism [27]. Differences were found on social impairments and developmental disturbances between the group of South Korean children and American children. It was concluded that these differences could be the result of differences in symptoms and not only the result of parental perception.

### 2.3. Implementing Screening for ASD

The foregoing discussion has implications for the assessment of autism in different cultures. First the tools used to screen for ASD need to be considered. As Triandis [11] noted, when cultures are very diverse, different assessment methods may have to be used in each culture. Since the identification of autistic disorder is based on parent response to an interview, an on-going study is analyzing the ADI-R item data for 250 Iranian children to establish the behaviours that are less present in Iranian children compared to British samples with a diagnosis of autistic disorder and to track the extent to which these items are included in the screening tool—the SCQ [15]. This may result in a revised screening tool for autism that is more sensitive to the Iranian culture. Meantime, in a comparative study performed in nine Arabic speaking countries [28], it was found that the Modified Checklist for Autism in Toddlers (M-CHAT) [29]—a popular screening tool in Western countries—is an applicable tool to be used for screening children for ASD in Arab countries.

A second consideration is the availability of trained personnel to oversee screening and diagnostic services. The Iranian data replicates a finding from the international literature of higher prevalence of ASD in more developed, urban areas. This is often attributed to better trained professionals who are more skilled in assessing children for ASD along with better access to child development services than is the case in less developed and rural regions [30]. Also parental literacy (illiteracy is more common in the rural areas in Iran) may affect parents' participation in screening and their engagement in face-to-face interviews. As the Iranian screening programme is extended to other Provinces and into rural areas, these service factors may need particular attention if uniform screening and assessment procedures are to be established nationally. Moreover, assessors from different disciplines should have opportunities to observe and interact with the children as part of the diagnostic process so as reduce the current reliance on parental reports.

Parental education is a further requisite to successful screening programmes in that parents need to be aware of the signs that their child may have a problem. Samadi et al. [31] interviewed 43 Iranian families who had a child with a confirmed diagnosis of ASD. All parents, except for one mother who was not so sure, thought that their children experienced problems. However, they generally described the children's difficulties as social communication problems with behavioural abnormality, and only one quarter of parents considered their child as having a learning disability. The absence of appropriate communication with the others is thus an important feature of ASD for the Iranian parent. But this perception could cause a delay in seeking a diagnosis of ASD for the child's lack of communication with others, instead of being recognised as a sign of a developmental problem, is often attributed to a personality trait in the child [2]. Moreover, communication deficits and behavioural problems cannot be fully observed until the child has the opportunity to communicate with others in different situations or environments outside of the family. Likewise, fixation with routines, another well-known ASD feature, tends to become obvious when the child is placed in a structured environment that does not let him show the behavioural pattern. Thus there needs to be a broader public education about children's development and potential signs of development problems.

In conclusion, screening for ASD at an early age is necessary internationally in order to confirm the suspicions and concerns that parents may have about their child's development and as a means of stimulating the provision of information and supports to families so that the children's development can be promoted. However, the tools used for screening need to be sensitive to the cultures in which they are used with training provided to the personnel available to undertake screening and accompanied by public education strategies around developmental disabilities such as ASD.

## 3. Parental Understanding of Autism

It has been noted that parents' need for information and advice is never more crucial than immediately after the diagnosis of autism [7]. This should include broad information about ASD in general such as its causes, alongside information on available therapeutic options and support services. Although the provision of such information in the affluent societies has a longer history compared to less affluent ones, research has shown that there is a general lack of information for parents of children with autism even in richer countries [32]. In absence of accurate information, parents may harbour false beliefs about the causes of ASD and whether or not anything can be done to help the child.

In Iran, Samadi et al. [31] interviewed individually at home 43 parents whose child had ASD ranging in age from 3 to 17 years with a mean of 8.2 years. They were recruited through special schools, centre and mother-child clinics. A structured interview schedule was followed, and in this section, we summarise the findings under five main themes and make comparisons with previous international research.

### 3.1. Parental Information Needs

Of the 43 parents interviewed, all but two (95%) wanted to get more information on ASD and its features. When parents were asked about their ideas as to the causes of their child's problem, their responses could be divided into six themes. These were as follows.

1. *Pregnancy: *Sixteen parents (*N* = 37%) attributed the cause to maternal factors during pregnancy such as maternal stress, complications of birth, and maternal cravings.
2. *Environmental factors: *Ten parents (23%) mentioned factors such as air pollution, chemicals, electromagnetic waves, and also the weapons used in Iran during the Iraq war.
3. *Damage to the child's brain or body: *A defect in the child's brain or body was mentioned by seven parents (16%).
4. *Spiritual and religious factors: *Five parents (12%) mentioned spiritual or religious causes. They talked about God's test and probable sins that they might have done unconsciously.
5. *Heredity (genetics): *Three parents (7%) mentioned a genetic link for their child's ASD.
6. *Lack of relationship with the others. *One parent talked about their family's special situation with minimal social contact with others and another about the lack of contact her son had with other people.

Finally, five parents (12%) had no idea about the causes or they said that they did not care about the causes whatever they were.

These findings differ markedly from present understanding as to the causes of ASD. Significantly only three (7%) of the Iranian parents mentioned a genetic link as the cause of ASD, whereas in a Canadian study with 41 parents, most (*N* = 37, 90%) believed that genetic reasons contributed to ASD in their children [33]. By contrast with a sample of 71 American parents of children with ASD [34], 16 (26%) considered there was a genetic predisposition to ASD but a larger group (*N* = 18, 29%) believed that immunizations were the cause of ASD. The latter was not mentioned by any Iranian parent although some did speculate on environmental influences. Also Iranian parents mentioned other causes for which there is little empirical evidence such as maternal factors during the pregnancy and spiritual or religious influences. Indeed the latter has been found to be a dominant explanation for disability in other nonwestern societies [35]. The attribution of disability to sins or immoral deeds committed by the afflicted person's family or even ancestors leads to cultural shame and the blaming of family members and individuals with disabilities, thereby threatening the cohesiveness of the family unit [36].

### 3.2. Sources of Information

When parents were asked about their sources of information on ASD, in the study by Samadi et al. [31] 10 of the 43 Iranian parents (23%) mentioned other parents who have a child with ASD. Books and printed materials were the second ranked source of information and were mentioned by nine parents (21%). Websites and the internet were mentioned by five parents (12%). This pattern is different from a study of 498 parents in the USA [37] who had a child with ASD. They reported the principal source of information as books (88%), websites (86%), and then other parents of children with ASD (72%).

In Iran, there are a limited numbers of published books on ASD in Farsi, and parental inability, or limited ability, in English language, makes web-based information and international books inaccessible sources of information. This may explain the greater dependency among Iranian parents to update their information through contacts with other parents, in clinic waiting rooms and while they are waiting to collect their children from school. Even so the proportions who did this were still small.

The danger of relying on other parents is that inaccurate information can be reinforced. Nevertheless, there is a powerful group experience in the oral tradition. Giving a speech or public speaking, by nature, is a group experience, whereas reading and writing tend to be a solitary experience. Although no study was found relating to preferred forms of Iranian communications, the importance of verbal communication for the Iranian has been noted as it engages them in different social activities and participating in group activities [38]. Although Iranians are different ethnic group to Arabs, as a group of Middle Easterns, they could have common features regarding their preferred communicational patterns [39].

One way forward then is to develop indigenous education courses on ASD to provide parents with the most up-to-date and reliable information. Trained parents in turn can transfer the information to other parents, a point we will come back to later. It also shows the importance of parental social networks and relationships with other parents with similar conditions in boosting their information on ASD. In a qualitative study undertaken on six trained mothers of the children with ASD in the USA [40], it was found that trained parents were easily able to gain the trust of the other parents of children with ASD and transfer information to them. Similar findings were found with American parents who attended a one week long educational programme [total 25 hours], and these parents were able in turn to train other parents [41].

In addition, other formats for sharing information need to be developed in local languages. A wide variety of methods have been documented, ranging from printed materials and verbal presentations, from discussions to audio visual materials alongside a variety of formats for sharing information with parents [42]. These included hand-outs with visual illustrations, videos, discussions of issues, exchanging experiences among parents, and letting parents' voices be heard.

### 3.3. What Parents Want to Know

Limited research has been undertaken into the topics that parents want and need to know about. Some general pointers can be drawn from the study undertaken in Iran by Samadi et al. [31]. The range of information should cover diverse parental needs in different stages. For instance, after a diagnosis of ASD for their child, parents' immediate reaction was shock and devastation. At that time they need to know about the causes, features of ASD, and the importance of the parental role in helping their child. They also need to learn how to cope with increased demands on them. Information about networking, advocacy, and support groups would also be helpful. Later on, information on how the child's development may be affected at different stages can be given alongside other topics such as, what it means to have ASD, the education options available, and useful teaching approaches.

Seven topics were identified in a factor analysis of American parents' responses to the information they had requested [8]. These topics were (a) how children develop; (b) how to play and talk with the child; (c) how to teach the child; (d) how to handle the child's behaviour; (e) information on the child's disability and conditions; (f) available services for the child; (g) services the child need in the future.

However, parents will need different information and support as their child grows older, such as information on sexual development and needs, vocational training needs for adolescents and young adults with ASD.

To conclude, in many countries, autism remains a mystery for many parents which is not surprising as it is a relatively newly identified condition. It is important that parents have access to accurate information and that this is made available in a range of formats that best suits them. This is a particular challenge in many developing countries with the lack of trained personnel and low levels of literacy.

### 3.4. The Impact on Parental Well-Being

Studies in various countries have shown that parents of children with ASD generally experience increased stress and have poorer health and family well-being [43–47]. That said, though there was wide variation among parents with some coping much better than others.

Having a child with ASD affects the health of Iranian parents, especially mothers. In the interviews with 43 parents [48], 32 of them 74% said that they do not feel well physically. Likewise their responses to the General Health Questionnaire [49] identified 29 parents [67%] as above the cut-off for poor health. Likewise in a Swedish study of 61 parents of children with ASD, it was found that they were less satisfied with their health compared to parents of non ASD children [50].

Mothers though are more prone to experience poorer health than fathers. In a study sample of 26 British mothers and 20 fathers of children with ASD, the mothers of children with ASD showed potentially higher levels of mental health problems [51]. This finding was replicated with Pakistani mothers of children with ASD who also showed higher rates of parental stress compared to fathers [52]. Likewise Iranian fathers as a group had lower levels of stress compared to a group of mothers [53].

It has been suggested that the difference of impact of ASD on parents could be due to gender roles connected to work and child rearing in most cultures [53]. According to Iranian culture, fathers are supposed to be strong and less emotional; they should be able to cope and handle different challenges for the family. Seventy-two percent of the fathers (*N* = 31) in an Iranian study [53] were the main wage earner of the family, while only one mother (2%) was the main wage earner and only eleven parents (26%) considered both fathers and mothers as the wage earners for the family. This responsibility could force fathers to devote their main energy to the financial situation of the family. By contrast, 79% of mothers (*N* = 37) said that they are considered to be the primary care provider for their children with ASD. There was only one father (2%) who was considered to be the main care provider, whereas 19% of parents (*N* = 8) said that both parents shared the responsibilities of care providing. Likewise in a study of 74 Chinese parents who took care of children with ASD, 45 mothers (61%) were considered as the main care provider for their child with ASD [54]. Except for three single mothers (7%), the rest of the families were two parents families and in these situations mothers had most of the responsibility for their child with ASD. These responsibilities varied from transportation and bringing the child to school or clinics to undertaking rehabilitative exercises as well as the ordinary duties of child rearing and household tasks.

Other international studies suggest that housewives are more vulnerable to health problems than mothers who were employed outside the home having better health [55]. One possible explanation could be that through work, mothers had wider social networks to receive support or information. Although parents may be reluctant to let others know about their child's ASD, having to work outside the house could improve their financial situation and also give them time for themselves to consider other issues rather than their child's ASD. These could contribute to employed mothers' improved health conditions.

Hence one explanation for maternal poorer health is that it results from a higher level of maternal responsibility in taking care of their child, combined with lack of informal and formal support to mothers [56, 57]; a theme we examine in the next section.

Finally one other cultural impact is worthy of note. Samadi [45] reported that parents of female children with ASD were more prone to health problems and to have elevated stress scores. Similar findings were reported for Pakistani parents, with parents of girls with ASD having higher stress scores compared to parents who had a boy with ASD [52]. These findings are different to those reported for British parents [51] which found no significant relationship between child gender and parental well-being. The difference here may derive from Iranian cultural beliefs which indicate that compared to boys; girls are more fragile and need more protection and help. Any type of disabilities may, therefore, increase the need for caring and supervision of the girls and place extra pressures on parents particularly in Middle-Eastern or Muslim countries.

### 3.5. Stress and Coping

In Samadi's Iranian study [45], a close relationship was also found between poorer health and reports of parental stress. In the interviews, he undertook with 43 parents, 37 (86%) parents reported themselves as stressed and 23 parents (53.5%) scored above the median on the Parental Stress Index used in this study. Moreover, it has been reported internationally that parents of children with ASD have higher levels of stress compared to parents whose children have other types of disabilities [58].

Hastings [59] suggested that much of the stressfulness of parenting a child with ASD comes from factors directly associated with the child's disorder such as their behaviour. In a study of 60 mothers and 8 fathers of children with ASD in the USA, it was found that the child's degree of severity of ASD symptoms were significantly correlated with parental level of stress [56]. Other studies have found that two main predictors of parental stress were the level of functional impairment and the presence of challenging behaviour in their children with ASD [60]. Indeed severity of behaviour problems exhibited by children and adults with developmental disabilities is one of the most significant stressors for family caregivers [56, 61].

Similar findings were reported in other studies when the child has intellectual disabilities. A cross-cultural study compared Irish, Taiwanese, and Jordanian mothers and found that behaviour problems exhibited by the child were correlated with maternal poorer well-being and stress [62]. Similar findings have been reported by researchers in other cultures such as Middle Eastern Arabs and English families [63, 64]. This suggests that the impact of a child's behavioural problem on parental stress and health problems is similar in different cultures. Moreover, daily stresses are important predictors of individual and family functioning. Families with higher degrees of daily stress tend to function less effectively [65].

The strategies used by families to cope with stress have not been well studied outside of western countries. Two forms of coping have been identified in the literature: namely, “problem-focussed coping” (finding strategies to deal with problem) and “emotional-focussed” coping (ignoring or wishing problems would go away.) In Samadi's [45] study of 43 parents, only 13 (30%) were categorised under the “problem-solving style” of coping and a larger number (*N* = 16, 37%) were categorised under “emotional style”. A statistically significant correlation was found between parental use of emotional style of coping strategies and poorer general health and with higher levels of parental stress. This replicates findings from British studies that also found that emotional-focused coping strategies were unhelpful to coping with demands associated with taking care of a child with ASD [64]. By contrast, parents who used rational coping strategies were more satisfied with their health, and they also showed lower levels of parental stress.

Samadi [45] also found no differences among mothers and fathers in their use of coping strategies but that older mothers used more problem-focussed coping strategies compared to younger mothers. This might indicate that mothers were able to draw on greater experience of dealing with their child with ASD. If so, they could then become a source of support to other mothers.

Another form of coping stems from parental perceptions of their role as carers. Samadi [45] found that the majority of parents claimed that they were satisfied with their caring role (*N* = 32, 74%). By contrast, the main reason given by eleven parents (26%) who said they were dissatisfied with their caring role was the pressures and troubles associated with parenting a child with ASD and also the lower cognitive abilities, which manifested itself in the low academic achievements of these children. A study in Turkey with 43 mothers of children with ASD reported that most mothers expressed feelings of burden and stress because of their child's behaviours associated with ASD [36].

Parents who expressed their satisfaction with their caring role generally said that they are satisfied because it was their duty to take care of their child regardless of the impact this might have on their lives. Taking care of children is an order from Allah to all Muslim parents. The words “children” and “child” are mentioned 118 times in the holy Quran in relation to different topics, but in Al-Nisa chapter, verse eleven, it is clearly mentioned as an order, “*Allah orders you concerning (the provision for) your children.*”

Having a child with different disabilities could be considered as a test from Allah to examine parents' faith from Iranian parents' point of view. Rejecting what has been given to them is called “Nashokri” or ingratitude, which causes Allah's wrath.

Likewise positive perceptions about caring can help parents to cope with the challenges and difficulties of bringing up a child with ASD in Iranian culture. It has been argued that the positive perceptions of parents about children with different severe forms of disabilities helped parents to cope with high levels of stress and served as an adaptive functioning [66]. This aspect might be worth exploring further with families internationally.

The wider cultural context can also be supportive of parents and this may be truer in nonwestern cultures. For instance, it has been noted that in the Turkish family system, the values of solidarity and mutual support are prevalent alongside cultural attitudes about resignation, particularly in the face of difficult events [36]. Sharing is also held in high regard in Turkish culture; people who have similar problems believe that their problems may be settled through sharing with and listening to others.

In summary, parents of children with ASD often experience poorer health and increased stress both of which could increase the strains they experience as parents. Parents may fall back on inappropriate coping strategies. However, cultural influences can impact positively as well as negatively on families, and these dimensions are worthy of further investigation.

## 4. Educating Parents about Autism in Developing Countries

Parents in many developing countries are reliant on informal sources of support as professional services are often poorly resourced with a lack of training and expertise in ASD among the available staff, be they doctors, teachers, or therapists. In time this state of affairs may change as societies become more affluent but even then, parents will continue to benefit from the informal supports provided by family and communities. Meantime whatever professional services exist within countries, they will need to harness local resources if families are to obtain the supports they require.

In this section, we examine the potential of providing education and training to groups of parents as a means of promoting the children's development and in meeting their personal needs as parents. This is a particularly cost-effective means of assisting families when professional resources are scarce as is the case in many low-income countries. Such programmes have been shown to be an effective method of support for parents and children with ASD in other countries as parents were able to develop skills and gain the necessary information to increase their children's communication skills and to decrease their challenging behaviours [67–70].

Although parent education programmes are long-established for children with intellectual disabilities, most of the evaluated programmes were prepared for Caucasian two-parent families who had sufficient financial resources and who were educated at the high school level [71]. This is unfortunate because parents without these qualifications need more support and education. Indeed parental culture could be an important issue for parent training programmes internationally as family services have to be more applicable to ethnic-minority populations. This requires the development of culturally modified parent education programmes as has happened for Asian parents whose children have intellectual disabilities [72]. However, parents in nonwestern countries have little opportunity to get information about ASD in any formal way.

Samadi et al. [73] developed and evaluated a seven-session, group-based course (around 10 hours in all) for Iranian parents with a focus on increasing their knowledge of ASD and boosting their coping strategies and well-being as parents. The content of the group sessions was determined from interviews conducted with the participating families (see earlier) but also informed by past literature and experiences in other countries. Each session covered a particular theme such as *What is ASD? Parental reactions to the diagnosis; myths and reality of ASD; society and ASD. *A package of audiovisual training materials, DVD clips and Powerpoint presentations, was assembled along with written materials that could be distributed to parents for further study at home but also as a resource that later could be disseminated to other organisations for use with their parents.

In each session, group discussions and activities were used to give the parents opportunities for sharing their ideas and thoughts with each other and to tell others about their personal stories and experience of ASD (but only if they are willing to do so).

### 4.1. Evaluating the Effectiveness of the Training

In all 37 parents of children aged 3 to 17 years participated in the training course (13 fathers and 24 mothers). They were divided into two groups, one of which received the training first while the other acted as a control group. The second group then also took part in the training which provided an opportunity to determine if it was effective with a second group of parents. Self-report rating scales of parental health, stress, family functioning, and coping strategies were completed before and after the training and then again some three months later to determine if any changes were maintained. The success of the training was evident in various ways [73].

- First no parents dropped out of the course and attendances were good: 26 parents (70%) attended all the training sessions, nine parents (24%) were absent from one session, and only two parents (5%) were absent from two sessions.
- Parents expressed satisfaction with the training course. All but one parent (97%) stated that they would recommend other parents of children with ASD to take part in similar training sessions. They were particularly pleased with the facilitator and the video clips they viewed.
- Sixteen parents (84%) in the first training group and 13 parents (72%) in the second training group reported that getting new information on children with ASD was the most important outcome of the training sessions for them.
- When the training sessions were finished for the first parental group, they showed significant improvements in their health, stress, and family functioning. Parents in the second group who had no training sessions showed no significant changes over the same period of time. However, when the second group received the training, their scores on these measures also showed significant changes. This suggests that the training had similar impact on them as it had with parents in Group 1.
- The parents in the first Group 1 maintained their improved scores or even improved on them—notably on ratings of stress and family functioning measures—even though they had not received any further training.
- Both groups of parents increased their use of rational coping strategies although there was no reduction in their use of emotional coping.

This study was different in two respects from the other studies done with parents of children with developmental disabilities in Iran and which might be deemed culturally inappropriate [74, 75]. The first difference was that the researcher met parents at their homes which helped to reduce the level of formality for the contacts and included fathers' ideas on ASD in that they were more likely to be at home as they often did not attend clinic appointments. The second difference was offering information in an informal friendly atmosphere with the aim of increasing the possibilities of more contacts between parents and to provide them with more opportunities to share their experience. Availability of refreshments in the sessions increased the level of informality and provided more opportunities for talking and exchanging experiences in the sessions. This had a further benefit in helping parents to create their own informal social networks during and after the training sessions.

This study demonstrates the impact that even a short but well-designed and structured training course can have on parental well-being. Other international studies have also found that parental education can reduce parenting stress although one study was done with only four mothers of children with developmental disabilities [76]. The improvement in family functioning may result from the opportunities parents were given to talk about their spouses and own experiences. All of these features could increase their level of understanding about their partners' feelings and improve their family functioning. The adoption of more rational coping strategies could result from parents having the opportunity to learn from one another and to form social support networks [77].

Although the findings of this study bode well for the future deployment of group-based support for parents in Iran and other less affluent countries, there are various challenges to making these types of programmes more widely available: the lack of the appropriately experienced facilitators, the dearth of family-centred approaches as practiced by the medical and educational services in Iran, and the recruitment of families from a broader range of socioeconomic backgrounds.

### 4.2. Parental Empowerment and Advocacy

In general terms, the educational sessions were aimed at empowering parents. This is a necessary goal for many reasons already stated, and this ethos needs to infuse all the supports that are provided to parents in whatever country of the world they live. It has been proposed that sharing skills and knowledge with families who have children with developmental disabilities is vital for empowerment [78]. Parents also need opportunities to exchange experiences and to be engaged with each other. They can then realise that they are not alone, and there are other parents with similar experiences “out there” [79]. Parents should be encouraged to feel they were part of a group as the feeling of solidarity can lead to greater advocacy.

Self-advocacy involves knowing when and how to approach others in order to negotiate desired goals, and in order to build better mutual understanding and trust, fulfilment and productivity [80]. A group consisting of members, who have similar experiences, provides support for each other [81] and membership of a self-help group can also lead to a new sense of self for parents of children with ASD [82]. In order to advocate for themselves, parents needed to discover common experiences, desires, hopes, and challenges. One pleasing consequence of the training study in Tehran was that a group of parents decided to form their own nongovernmental organisation for ASD, the first of its kind in Iran.

To summarise, a key priority in less affluent countries is the provision of education and training for families not only for the benefits it can bring to them but as a means of reaching out to many more parents as they pass on their knowledge and experiences to others through informal contacts. The formation of local associations will also promote parental empowerment, an ethos of self-help, and greater advocacy for the creation of more formal support services to assist the children and families.

## 5. Conclusions

As we have noted throughout, the main themes reviewed in this paper are not unique to the less developed nations of the world but they do pose particular challenges for families living in these countries and for how support services can be developed. Moreover, further indigenous research, albeit within a cross-cultural context, is essential if international understanding of Autism is to be advanced. This is pertinent to high income countries too because as immigrants arrive from other cultures, then in future their services will also require to become more culturally sensitive with respect to the supports provided to families [83].

The numbers of children diagnosed with ASD is likely to rise in coming years throughout the world but as yet most families, and indeed the general public, remain ignorant about the implications of this diagnosis and the likely impact it will have on the child's development and on family life. The provision of accurate information through accessible media and local languages is essential as are the opportunities for families to receive practical and emotional support as a means of reducing the stress and strain they will invariably experience. Internationally the emphasis has to be on empowering families and making them more resilient. Certainly in less developed countries with their dearth of professional services, self-help becomes the major strategy for on-going parental support, but awareness about how this can be fostered is poorly promoted [84]. Meeting the needs of families in rural communities requires particular attention.

A second major lesson is the need to view autism within a cultural context and not just as a medical condition requiring standard interventions. Hence a deeper understanding is needed as to how differences in children's development are viewed within and across cultures coupled with the explanations that families and the wider communities hold for these differences and how cultural beliefs can best be reformed in light of accruing knowledge. An essential part of this process means gaining a fuller understanding as to how informal and formal supports can be best provided to families that respect yet also seek to transform cultural practices that will enhance the quality of life of the child and of the family. The training of existing personnel in education, health, and social services on ASD awareness should be an immediate priority as they are well placed to provide initial advice and guidance to families [85]. In sum, the means for furthering international dialogue and cooperation must to be a priority in the coming years.
